# Endoscopic retrieval of an *N* -butyl-2-cyanoacrylate cast from the common bile duct: a rare complication of portal vein embolization

**DOI:** 10.1055/a-2864-1266

**Published:** 2026-05-21

**Authors:** Katsuhiko Sato, Teppei Yoshioka, Hiroki Satomura, Yasuharu Kawamoto, Hirofumi Akita, Hidetoshi Eguchi, Takahiro Kodama

**Affiliations:** 1 Department of Gastroenterology and Hepatology 38637 The University of Osaka Graduate School of Medicine Faculty of MedicineSuitaJapan; 2 Department of Radiology 38637 The University of Osaka Graduate School of Medicine Faculty of MedicineSuitaJapan; 3 Department of Gastroenterological Surgery 38637 The University of Osaka Graduate School of Medicine Faculty of MedicineSuitaJapan

Percutaneous transhepatic portal vein embolization (PTPE) is performed before hepatic resection to increase future liver remnant volume, and *N* -butyl-2-cyanoacrylate (NBCA) is widely used for PTPE. To our knowledge, this is the first report of a mobile NBCA cast in the bile duct successfully removed en bloc by endoscopic retrograde cholangiopancreatography (ERCP) after inadvertent migration during PTPE ( Video 1 ).


Endoscopic retrieval of a mobile NBCA cast from the bile duct using balloon sweeping following PTPE. NBCA, *N* -butyl-2-cyanoacrylate; PTPE, percutaneous transhepatic portal embolization.
Video 1

**Fig. 1:**
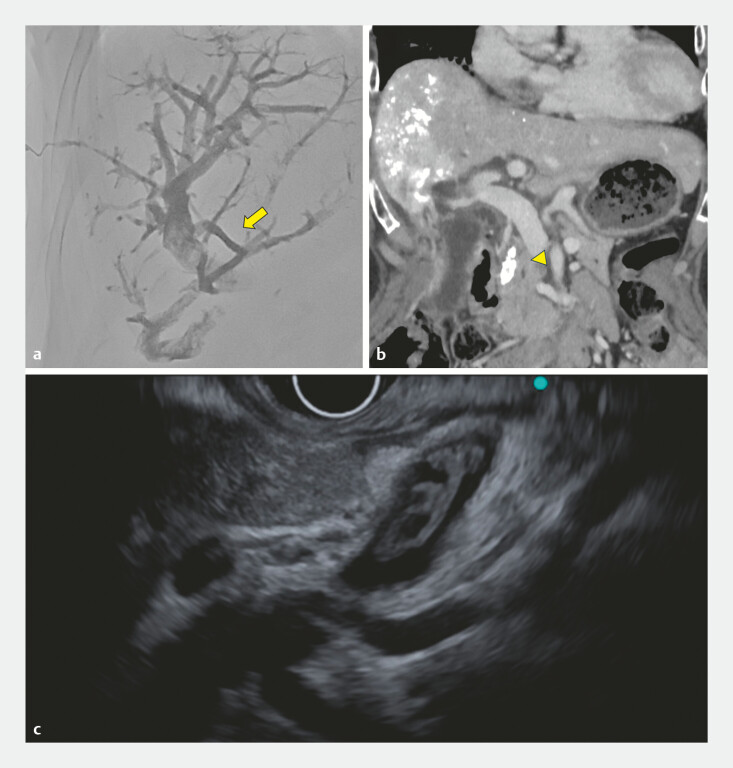
An embolic material after percutaneous transhepatic portal embolization (PTPE). **a** Inadvertent anterior bile duct embolization with *N* ‑butyl‑2‑cyanoacrylate (NBCA; arrow). **b** Portal‑phase CT showing distal bile duct migration (arrowhead). **c** Endoscopic ultrasound revealing a mobile tubular structure in the common bile duct. CT, computed tomography.

**Fig. 2:**
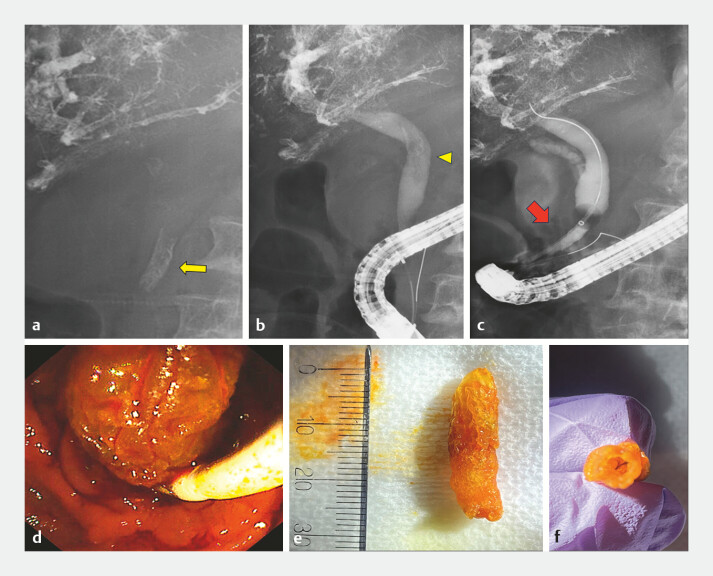
ERCP findings and the retrieved embolic material. **a** Distal bile duct localization (yellow arrow). **b** Proximal migration during cannulation (yellow arrowhead). **c** Endoscopic balloon removal (red arrow). **d** Tubular fragment retrieved via papilla. **e** 25 mm, yellow, rough surface. **f** Hollow, straw-like interior. ERCP, endoscopic retrograde cholangiopancreatography.

We report a 75-year-old woman with intrahepatic cholangiocarcinoma. Right hepatectomy was planned, but insufficient remnant liver volume required PTPE. During NBCA–Lipiodol tract embolization, inadvertent bile duct puncture caused NBCA migration into the anterior branch ( Fig. 1 **a** ). One week later, persistent liver dysfunction and computed tomography revealed a cast-like embolic material causing bile duct obstruction ( Fig. 1 **b** ). Although NBCA adhesion to the bile duct wall was a concern, endoscopic ultrasound (EUS) showed a freely mobile intraductal cast ( Fig. 1 **c** ), so ERCP removal was chosen. ERCP with balloon dilatation and extraction successfully removed material en bloc ( Fig. 2 **a–d** ). Because the liver became ischemic after PTPE, endoscopic sphincterotomy was avoided to reduce the risk of retrograde infection and hepatic abscess. The retrieved structure was tubular and non-adhesive ( Fig. 2 **e, f** ). Follow-up imaging confirmed complete removal, and the patient had an uneventful recovery.

NBCA is mainly used for vascular embolization and is effective in PTPE, with a 23% complication rate, primarily abdominal pain and hepatic dysfunction 1 . Although this occurred unintentionally, no reports exist for direct NBCA injection into a normal bile duct, intraductal mobility, or ERCP removal. Regarding intraductal NBCA use, only a few reports describe postoperative biliary fistula embolization, and the procedure is considered feasible and safe 2 3 .

This case highlights obstructive jaundice caused by NBCA migration into the bile duct following PTPE. When embolic mobility is confirmed by EUS, ERCP with balloon extraction provides a safe, minimally invasive alternative to percutaneous transhepatic biliary drainage or extrahepatic bile duct resection, despite risks such as pancreatitis and distal impaction.

Endoscopy_UCTN_Code_CPL_1AK_2AF
